# Pollen-Food Allergy Syndrome: From Food Avoidance to Deciphering the Potential Cross-Reactivity between Pru p 3 and Ole e 7

**DOI:** 10.3390/nu16172869

**Published:** 2024-08-27

**Authors:** Paula Álvarez, Rocío Aguado, Juan Molina, Antonio Trujillo-Aguilera, Mayte Villalba, Araceli Díaz-Perales, Carmen Oeo-Santos, Eduardo Chicano, Nadine Blanco, Ana Navas, Berta Ruiz-León, Aurora Jurado

**Affiliations:** 1 Department of Immunology and Allergy, Reina Sofía University Hospital, 14004 Córdoba, Spain; z92alrop@uco.es (P.Á.); rocio.aguado.alvarez.sspa@juntadeandalucia.es (R.A.); trujillo_1993@hotmail.com (A.T.-A.); nadine.blanco.sspa@juntadeandalucia.es (N.B.); mb.ruiz.sspa@juntadeandalucia.es (B.R.-L.); aurora.jurado.sspa@juntadeandalucia.es (A.J.); 2 Maimonides Biomedical Research Institute of Córdoba (IMIBIC)/Reina Sofía University Hospital, University of Córdoba, 14004 Córdoba, Spain; eduardo.chicano@imibic.org; 3 Allergy Network ARADyAL, Instituto de Salud Carlos III, 28029 Madrid, Spain; mvillalba@quim.ucm.es (M.V.); araceli.diaz@upm.es (A.D.-P.); 4 Department of Biochemistry and Molecular Biology, Faculty of Chemical Sciences, Complutense University of Madrid, 28040 Madrid, Spain; 5 Centre for Plant Biotechnology and Genomics (CBGP, UPM-INIA), Polytechnic University of Madrid, 28223 Madrid, Spain; 6 Department of Physiology, Biochemistry and Human Genetics, Faculty of Health Science, Rey Juan Carlos University, 28922 Madrid, Spain; carmenoeos@gmail.com; 7 IMIBIC Mass Spectrometry and Molecular Imaging Unit (IMSMI), 14004 Córdoba, Spain

**Keywords:** food allergy, pollen-food syndrome, cross-reactivity, epitope mapping, Ole e 7 allergen, Pru p 3 allergen, nonspecific lipid transfer proteins (nsLTPs)

## Abstract

Background: Cross-reactivity between nonspecific lipid transfer proteins could cause anaphylaxis, further influencing food avoidance and nutrient deficiencies. The one affecting olive pollen (Ole e 7) and peach (Pru p 3) may underlie a variety of pollen-food syndromes, though a deep molecular analysis is necessary. Methods: Three Ole e 7-monosensitised patients (MON_OLE), three Pru p 3-monosensitised patients (MON_PRU) and three bisensitised patients (BI) were selected. For epitope mapping, both digested proteins were incubated with patient sera, and the captured IgE-bound peptides were characterised by LC-MS. Results: The analysis revealed two Ole e 7 epitopes and the three Pru p 3 epitopes previously described. Interestingly, the “KSALALVGNKV” Ole e 7 peptide was recognised by MON_OLE, BI and MON_PRU patients. Conversely, all patients recognised the “ISASTNCATVK” Pru p 3 peptide. Although complete sequence alignment between both proteins revealed 32.6% identity, local alignment considering seven residue fragments showed 50 and 57% identity when comparing “ISASTNCATVK” with Ole e 7 and “KSALALVGNKV” with Pru p 3. Conclusions: This study mapped sIgE-Ole e 7-binding epitopes, paving the way for more precise diagnostic tools. Assuming non-significant sequence similarity, structural homology and shared key residues may underlie the potential cross-reactivity between Ole e 7 and Pru p 3 nsLTPs.

## 1. Introduction

Food allergy is an aberrant, potentially life-threatening immune response induced by exposure to a harmless food antigen [1]. Food allergy represents a growing socioeconomic and public health problem, with an increasing prevalence in the last two decades. Worldwide, its prevalence is estimated to range from 0.45% to 10% in children under five years. Sensitisation to nonspecific lipid transfer proteins (nsLTPs), particularly from the Rosaceae family, such as those in peaches, is becoming a clinical concern in Mediterranean countries, representing the most prevalent cause of food allergies [1,2]. It is common for patients referred for consultation to have reacted to one or a few allergenic sources, but they are frequently polysensitised to multiple LTPs. In clinical practice, the finding of sensitisation to different LTPs (LTP syndrome) implies a challenge for allergists. In food allergy, treatment relies on avoiding the allergen causing the reaction [1,3,4,5]. However, this can be thwarted in the case of accidental exposure to a rare or hidden allergen, leading to a monotonous diet and a consequent reduction in the quality of life of the patients and their families [3]. Food allergies have a major impact on child health and growth due to inadequate nutrient intake, and the influence on growth and nutrient deficiencies is more pronounced in children with multiple food allergies [5].

The pollen-food syndrome is characterised by allergic manifestations following the ingestion of food in patients sensitised to pollen [1,6]. Additionally, the geographical distribution of food allergy to nsLTPs follows a north–south gradient, with symptoms particularly severe in southern countries. This is reflected in countries such as Spain or Italy, where sensitisation to Pru p 3 reaches 9–12% of allergic patients, of which almost half have reported anaphylactic systemic reactions, whereas, in Central and Northern Europe, most cases of nsLTP sensitisation are clinically irrelevant and focused on other nsLTPs [6]. This has prompted investigations into a seasonal aeroallergen as a potential primary sensitiser able to trigger LTP syndrome through a cross-reactivity phenomenon. Among respiratory allergens, olive tree (*Olea europaea*) pollen is a major contributor to allergies across the Mediterranean [7]. This is evident in regions of Spain, like Andalusia, or Italy (Apulia and Campania), where olive tree farming is massive [8,9,10]. Among the olive allergenic proteins identified so far, the nsLTP Ole e 7 emerges as the main allergen responsible for severe asthmatic symptoms in regions with a high environmental presence of olive tree pollen, despite being categorised as a relatively minor allergen [8,9,11,12]. Interestingly, patients sensitised to Ole e 7 in these regions often exhibit allergic symptoms to peaches (*Prunus persica*) or co-sensitisation to the peach nsLTP Pru p 3 [13,14], suggesting a potential linkage between these nsLTPs within the same geographical framework. Even more, the overall prevalence of Pru p 3 sensitisation in the Spanish pollen-allergic population was 12.6% [6]. In the database of our unit, set in a geographical environment that supports olive pollen counts over 5000 grains/m^3^ [14], laboratory requests for specific IgE (sIgE) to Ole e 7 or Pru p 3 are very frequent, especially for Ole e 7. The positivity rate is 72% for Ole e 7 and 17% for Pru p 3, including all the requests from the allergy department. When considering only the samples where both allergens are requested, the combined positivity rate for Ole e 7 and Pru p 3 rises to 52% (healthcare data). This indicates that more than half of patients tested for these two nsLTPs are co-sensitised.

Cross-reactivity between proteins occurs when an antibody directed against a specific antigen can recognise a different one because of a similar structure [15,16]. This is the case of phylogenetically related allergens that trigger the pollen-food syndrome [6,17]. Numerous studies have demonstrated cross-reactivity between pollens and foods, including nsLTPs from different species [18,19,20,21,22,23,24,25,26,27]. Nevertheless, it tends to be more challenging to explain between unrelated ones. In Southern Europe, Pru p 3 is considered the primary sensitiser, triggering the LTP syndrome in other food allergenic proteins with commonalities in their structure [28,29,30]. Even so, the coexistence of sIgE sensitivity to Pru p 3 and Ole e 7 in these regions has brought attention [14]. However, despite both allergenic proteins belonging to the same biological family, they lack apparent commonalities that would explain the cross-reactivity. To date, there is no reliable evidence supporting this dual sensitisation, nor the natural progression of sensitisation from the primary sensitiser to other nsLTPs, leaving a gap in our understanding of the potentially severe sensitisation observed in some Mediterranean regions. Cross-reactivity between nsLTPs could trigger severe allergic reactions, leading to a restricted diet with a negative impact on allergic individuals and their families. Therefore, identifying and characterising allergy-relevant epitopes is crucial for allergy diagnosis, immunotherapy implementation and nutritional deficiencies prevention.

In this context, the development of recombinant technology has enabled the production of allergens as recombinant proteins that retain the structural and functional properties of the natural allergen [31,32,33,34,35]. New molecular strategies have been explored to identify regions with IgE-binding capacity [36,37,38,39,40].

This study has the dual objective of mapping, for the first time, IgE-binding linear epitopes from Ole e 7 and then identifying cross-reactive IgE-binding linear epitopes between these phylogenetically distant molecules, Ole e 7 and Pru p 3. For this purpose, two novel immunocapture approaches and liquid chromatography coupled to a high-resolution mass spectrometer were used.

## 2. Materials and Methods

### 2.1. Patient Selection

This cross-sectional observational study was conducted according to the relevant national regulations, institutional policies and the tenets of the Helsinki Declaration and was approved by the Ethics Committee of the Reina Sofia University Hospital (ref. 4508). Written informed consent was obtained from all participants, and all samples were handled anonymously. Patients were recruited from the Immunology and Allergy Department based on (1) a confirmed history of allergy to olive pollen or peaches, (2) the presence of sIgE to Ole e 7 or Pru p 3 and (3) a positive Basophil Activation Test (BAT) to Ole e 7 or Pru p 3.

In a previous study by our group, four out of thirteen patients (30%) sensitised to Ole e 7 in the absence of sIgE to Pru p 3 showed a positive BAT result using recombinant Pru p 3 as a stimulus besides rOle e 7 [14]. Therefore, we deliberately selected three of these patients to investigate the potential causes of this phenomenon. Additionally, to assess the recognition capability of these proteins in allergic individuals in both directions, we included three patients with sIgE to Pru p 3 without sensitisation to Ole e 7 who degranulated in the presence of rPru p 3 and three patients sensitised to Ole e 7 and Pru p 3 who degranulated in the presence of both allergens. Individuals who underwent immunotherapy in the five years preceding the study were excluded. The clinical assessment included a thorough examination of the patient history and a skin prick test. The skin prick test was performed by the European guidelines [41] using commercial extracts from *Olea europaea* pollen and peaches (ALK Abelló). Due to the reported heterogeneous IgE reactivity of the patients [14], we used a sera pool from each patient group (MON_OLE, MON_PRU and BI) for the immunocapture. A serum sample from a non-allergic individual was used as a negative control.

### 2.2. sIgE nsLTP Profile

The sIgE to Ara h 9 (peanut nsLTP), Art v 3 (mugwort nsLTP), Cor a 8 (hazelnut nsLTP), Jug r 3 (walnut nsLTP), Mal d 3 (apple nsLTP), Ole e 1 (olive pollen major antigen), Ole e 7 (olive pollen nsLTP), Pru p 3 (peach nsLTP), Par j 2 (parietaria nsLTP) and Tri a 14 (wheat nsLTP) was measured using ImmunoCAP 250 (Thermo Fisher Scientific^TM^, Uppsala, Sweden) following the manufacturer’s recommendations. Levels > 0.35 kU/L were considered positive. Accordingly, the patients were categorised into three groups based exclusively on their Ole e 7 and Pru p 3 sIgE profiles: MON_OLE patients had sIgE to Ole e 7 > 0.35 kU/L and sIgE to Pru p 3 < 0.35 kU/L, MON_PRU patients had sIgE to Pru p 3 > 0.35 kU/L and sIgE to Ole e 7 < 0.35 kU/L and BI patients had a sIgE level > 0.35 kU/L to both allergens. The presence of sIgE against other allergens was not taken into account in this classification.

### 2.3. Proteins, Reagents and Antibodies

Recombinant Ole e 7 and Pru p 3 were generated following established protocols [14,31,42]. Sequence-grade trypsin was acquired from Promega Biotech Corporation^TM^ (Madison, WI, USA), while mouse anti-human purified IgE antibody was obtained from Bio-Rad^TM^ Laboratories Inc. (Hercules, CA, USA). Additionally, peroxidase-labelled goat anti-human IgE was sourced from Sigma-Aldrich^TM^ (St. Louis, MO, USA).

### 2.4. Basophil Activation Test

The release of mediators of the allergic response induced by rOle e 7 and rPru p 3 was analysed in basophil cells. Previously, a dose–response curve was performed, and the best concentration for specific degranulation was established at 10 μg/mL of each protein. Therefore, BAT was performed using rOle e 7 or rPru p 3 at 10 μg/mL, respectively, as the stimuli, as previously described [14]. PBS was used as a negative control and N-formyl-methionyl-leucyl-phenylalanine (fMLP, ref. f3506, Sigma-Aldrich) as a positive control. Cell staining was performed using an anti-human CD63 FITC/CD123 PE/HLA-DR PerCP antibody cocktail (ref. 341068, BD FastImmune^TM^, Becton, Dickinson and Company, San Jose, CA, USA). The acquisition was performed in a BD FacsCanto II cytometer (Becton Dickinson and Company, San Jose, CA, USA), using BD FacsDiva^TM^ version 8.0.1 as the acquisition software and Kaluza Analysis 2.2 as the analysis software. At least 500 CD123^+^ events were recorded. Basophil degranulation was measured as the percentage of basophils expressing CD63 (%CD63^+^) (Supplementary Materials Figure S1).

### 2.5. Epitope Mapping

**Figure 1:**
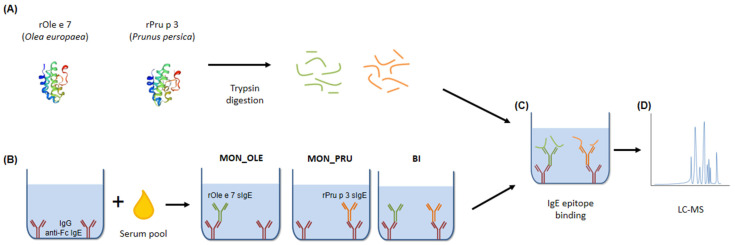
Schematic diagram of the novel method used for epitope mapping. (**A**) Recombinant Ole e 7 and Pru p 3 were digested with trypsin. (**B**) ELISA plate was coated with IgG anti-Fc IgE antibodies and incubated with sera from bisensitised (BI), Ole e 7 monosensitised (MON_OLE) or Pru p 3 monosensitised (MON_PRU) patients; a serum sample from a non-allergic individual was employed as a negative control. (**C**) Digested proteins were then added to corresponding wells. Bound peptides recognised by sIgE were subsequently eluted with trifluoroacetic acid and preserved. (**D**) Finally, rOle e 7 and rPru p 3 peptides were analysed by LC-MS.

The epitope mapping procedure, schematised in Figure 1, is described briefly below and in more detail in Section 2.5.2.

#### 2.5.1. Enzymatic Digestions

A total amount of 20 µg of each purified recombinant protein was digested by adding 50 mM dithiothreitol in ammonium bicarbonate and incubating at 60 °C for 30 min. This was followed by a second step with 110 mM iodoacetamide at room temperature (RT) for 30 min and a final incubation with 0.2 µg/µL of MS sequencing grade trypsin at 37 °C for 17 h in a final volume of 90 μL. The digestion was stopped by adding 10% trifluoroacetic acid at 37 °C for 1 h, reaching a pH of 2.5. After centrifugation, the supernatant was collected, and 100% acetonitrile and 25 mM ammonium bicarbonate were added. Two more centrifugations at 4 °C were performed to ensure that the cloudy material was completely removed.

#### 2.5.2. Immunocapturing of Specific Epitope Peptide Fragments

The serum IgE capture was conducted using the protocol described by Chen et al. for ELISA assays [43], with in-house modifications. In brief, 96-well plates (Corning^®^ 96-Well EIA/RIA Assay Microplate with a high binding surface; Merck KGaA, Darmstadt, Germany) were coated overnight at 4 °C with 0.2 µg/well (1 µg/mL) purified mouse anti-human IgE. Then, the plates were blocked with 3% skim milk in PBS and incubated for 1 h at RT. The plates underwent four washes with 0.05% Tween 20 between each step. The sera pool from each group of allergic subjects was diluted 1:10. Then, 100 μL per well of each pool (MON_OLE, MON_PRU and BI) were separately added to the plate and incubated for 2 h at RT. Subsequently, the plates were washed twice with PBS, and 6 μg of Ole e 7 or Pru p 3 peptides, obtained in Section 2.5.1, were added to the corresponding well and incubated for 2 h at RT, according to the following scheme:

- MON_OLE with 6 μg of Ole e 7 peptides
- MON_PRU with 6 μg of Ole e 7 peptides
- BI with 6 μg of Ole e 7 peptides
- MON_OLE with 6 μg of Pru p 3 peptides
- MON_PRU with 6 μg of Pru p 3 peptides
- BI with 6 μg of Pru p 3 peptides.

Following three washes with PBS, the immune-specific captured peptides were eluted twice with 0.2% trifluoroacetic acid (100 µL, 15 min, RT). Serum from a non-allergic individual was used as a negative control.

#### 2.5.3. LC-MS Analysis (Liquid Chromatography Coupled to Mass Spectrometry)

Following the immunocapture, the affinity-bound peptides were characterised using LC-MS following the outlined protocol.

##### Sample Preparation

Following the manufacturer’s instructions, a total volume of 20 µL of the recovered peptides eluted with trifluoroacetic acid was loaded onto Evotips (Evosep, Odense, Denmark) [44]. Additionally, 200 ng of Pierce™ HeLa Tryptic Digest Standard (Thermo Scientific, Waltham, MA, USA) was used as the quality control and system equilibration.

##### Liquid Chromatography

The purified peptides underwent separation using the predefined 60 SPD method (21-min gradient time) on an Evosep One LC system (Evosep) [45]. A fused silica 10-μm ID emitter (Bruker Daltonics, Billerica, MA, USA) was positioned within a nanoelectrospray source (CaptiveSpray source, Bruker Daltonics). The emitter was linked to an 8-cm × 150-μm reverse-phase column filled with 1.5-μm C18 beads (EV1109). The column was maintained at a temperature of 40 °C in an oven compartment (Bruker Daltonics). The mobile phases employed were 100% water and 100% acetonitrile, both buffered with 0.1% formic acid (LC-MS grade, Fisher Scientific, Hampton, NH, USA).

##### Mass Spectrometry

The liquid chromatography was coupled online to a TIMS Q-TOF instrument (timsTOF Pro, Bruker Daltonics) using the Data-Dependent Acquisition-Parallel Accumulation Serial Fragmentation (DDA-PASEF) method through a CaptiveSpray nanoelectrospray ion source [46]. In the acquisition mode, the ion mobility dimension underwent calibration with three Agilent ESI-L Tuning Mix ions (*m*/*z*, 1/K0: 622.0289 Th, 0.9848 Vs cm^−2^; 922.0097 Th, 1.1895 Vs cm^−2^; 1221.9906 Th, 1.3820 Vs cm^−2^). Additionally, the collision energy experienced a linear decrease from 59 eV at 1/K0 = 1.6 Vs cm^−2^ to 20 eV at 1/K0 = 0.6 Vs cm^−2^. The DDA-PASEF method employed the “long gradient method” (1.1 s cycle time), with the accumulation and ramp times set to 100 ms. Singly charged precursors were excluded from fragmentation using a polygon filter in the (*m*/*z*, 1/K0) plane. Moreover, all precursors reaching the target value of 20,000 were excluded for 0.4 min. The precursors were isolated using a Q window of 2 Da for *m*/*z* < 700 and 3 Da for *m*/*z* > 800.

##### Data Analysis

The sample acquired in DDA-PASEF mode was used for data analysis and protein identification. The computational platform FragPipe (version 17.1), including MSFragger, was employed to accomplish this. Peptide identification from tandem mass spectra (MS/MS) was carried out using the MSFragger search engine, with raw (.d) files serving as the input. An in-house sequence database was constructed to narrow the search focus to the target peptides. Protein sequences were extracted from UniProt and the study by Oeo-Santos et al. [31,47]. Reversed protein sequences were added to the original databases as decoys. In the MSFragger analysis, both the precursor and (initial) fragment mass tolerances were configured to 20 ppm. Spectrum deisotoping [48], mass calibration and parameter optimisation 8/10/10 were activated. Enzyme specificity was defined as “trypsin”, and fully enzymatic peptides were permitted. Up to two missed trypsin cleavages were allowed. The isotope error was set to 0/1/2. The peptide length ranged from 7 to 50, and the peptide mass spanned from 500 to 5000 Da. Variable modifications included the oxidation of methionine, acetylation of protein *N*-termini, −18.0106 Da on *N*-terminal glutamic acid and −17.0265 Da on *N*-terminal glutamine and cysteine. Carbamidomethylation of cysteine was designated as a fixed modification. The maximum number of variable modifications per peptide was set to 3.

For each analysis, the MS/MS search results underwent additional processing using the Philosopher toolkit [49]. Initially, MSFragger output files (in pepXML format) were processed using PeptideProphet with high-mass accuracy binning and semi-parametric mixture modelling options [50], calculating the posterior probability of the correct identification for each peptide-to-spectrum match (PSM). The resulting output files from PeptideProphet (also in pepXML format) were then processed using ProteinProphet to assemble peptides into proteins (protein inference) and to generate a combined file (in protXML format) containing high-confidence protein groups, along with the corresponding peptides assigned to each group [51]. The combined protXML file underwent further processing using the Philosopher Filter module following these steps: each identified peptide was categorised either as a unique peptide assigned to a specific protein (or protein group containing indistinguishable proteins) or assigned as a razor peptide to a single protein (protein group) that possessed the most peptide evidence. The protein groups assembled by ProteinProphet, with the probability of the best peptide used as the protein-level score, were subjected to filtering at a 1% protein-level False Discovery Rate (FDR) using the picked FDR strategy considering both unique and razor peptides [52,53].

The final reports were generated and filtered at each level (PSM, ion, peptide and protein) using the 2D FDR approach (1% protein FDR plus 1% PSM/ion/peptide-level FDR for each corresponding PSM.tsv, ion.tsv and peptide.tsv file) [54]. For each analysis, the “*.pepXML” files were submitted to TransProteomicPipeline (http://www.tppms.org/ (accessed on 23 November 2021)) to verify the presence or absence of the target peptides. The quantitation of each peptide was expressed as intensity (in arbitrary units from raw data) and ions (number of ions matched for each detected peptides).

### 2.6. Protein and Peptide Sequence Alignment

The sequence analysis of the proteins and peptides recognised by each group of patients was performed using EMBOSS Needle for global alignment in the case of whole protein sequences (UniProt code Q9LED1 for Pru p 3 and Ole e 7 sequence from Oeo-Santos et al. [31]) and SIM for local alignment of the peptides obtained in the mapping [55,56].

### 2.7. Automated Search for Homologous Sequences

The search for homologous sequences was performed using open-access algorithms on the NCBI web portal [57].

## 3. Results

### 3.1. Patient Characteristics

A total of nine patients were enrolled. All MON_OLE patients experienced rhinoconjunctivitis and bronchial asthma due to the exposure to olive pollen, whereas all MON_PRU patients reported allergic reactions following the ingestion of Rosaceae fruits. In addition, MON_PRU patients exhibited respiratory symptoms. As expected, BI patients showed both oral and respiratory symptoms. Concerning the complete nsLTP sIgE profile studied (Table 1), this included food allergens (Pru p 3 from peach, Mal d 3 from apple, Tri a 14 from wheat, Jug r 3 from walnut, Cor a 8 from hazelnut and Ara h 9 from peanut, all more or less related to Pru p 3) and also aeroallergens (Ole e 7 from olive pollen, Art v 3 from mugwort and Par j 2 from parietaria). Furthermore, the major allergen of olive pollen, Ole e 1, was included in patients sensitised to olive pollen. MON_OLE patients did not show sensitisation to any other nsLTP. However, both MON_PRU patients showed sensitisation to almost all the nsLTPs studied, except for Par j 2 and Ole e 7. BI patients had circulating sIgE to the majority of nsLTPs.

### 3.2. rOle e 7 and rPru p 3 Epitope Mapping

The LC-MS analysis revealed two rOle e 7 epitopes (Table 2):

O1, residues 10–22: KLTSCVSYLDDKS
O2, residues 54–64: KSALALVGNKV

**Figure 2:**
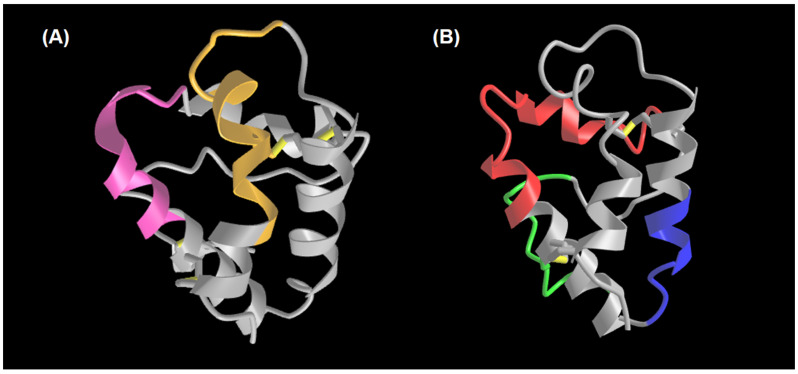
Ole e 7 and Pru p 3 proteins and the mapped epitopes. (**A**) Three-dimensional structure of Ole e 7 predicted by ESMFold using the protein sequence described by Oeo-Santos et al. [31] and visualised by iCn3D viewer. The orange and pink fragments represented O1 and O2, respectively. (**B**) The structure of Pru p 3, visualised by iCn3D viewer using Protein Data Bank code 2ALG. Blue, red and green fragments corresponded to the P1, P2 and P3 epitopes, respectively.

The MON_OLE sera pool recognised both peptides; meanwhile, BI and MON_PRU patients specifically recognised the O2 peptide. rOle e 7 epitopes resulting from the mapping were overlapped in a predicted three-dimensional structure of the complete protein (Figure 2A).

In a similar process for the rPru p 3 protein, epitope mapping revealed three peptides:

P1, residues 33–39: NVNNLAR
P2, residues 53–72: QLSASVPGVNPNNAAALPGK
P3, residues 81–91: ISASTNCATVK

Bisensitised patients recognised all three peptides, while MON_PRU and MON_OLE patients specifically recognised the P3 peptide. These epitopes were highlighted in the already-known structure of Pru p 3 (Figure 2B).

The epitope mapping output data, including the quantification of each recognised peptide, are shown in Table S1 in the Supplementary Materials section.

### 3.3. Sequence and Structural Alignment Comparison

**Figure 3:**
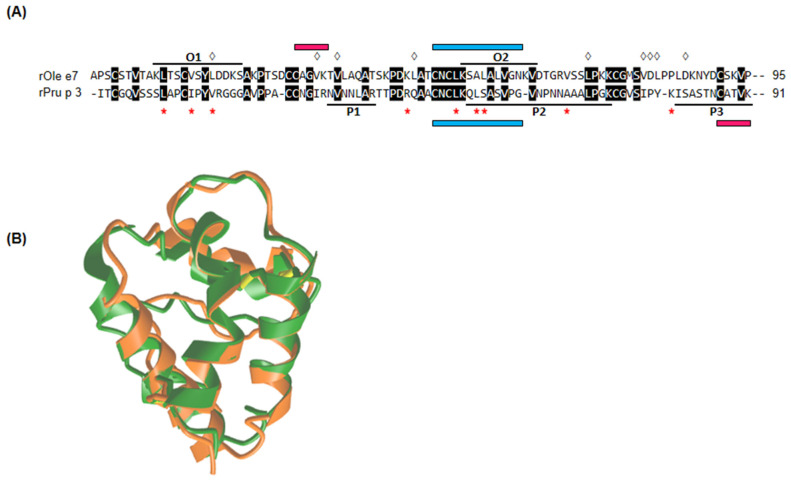
Sequence and structural alignment comparison. (**A**) Alignment of the amino acid sequences of Pru p 3 (UniProt code Q9LED1) and Ole e 7 [31], highlighting with a black line the IgE-binding peptides recognised by all groups of patients. ◊: Ole e 7 residues associated with oleic acid binding, *: Pru p 3 residues associated with lipid binding, blue lines represented Ole e 7 sequences potentially associated with cross-reactivity and pink lines represented Pru p 3 sequences potentially associated with cross-reactivity. (**B**) Structural alignment of the predicted Ole e 7 (green) and Pru p 3 (orange) structures by TM-align; TM-score: 0.8574.

The sequence alignment of Pru p 3 and Ole e 7 revealed a sequence identity of 32.6% between both proteins (Figure 3A). The structural comparison between both proteins is shown in Figure 3B. The unaligned comparison of the P3 peptide with Ole e 7 and O2 peptide with Pru p 3 showed an identity between 50 and 57% for fragments containing at least seven residues. Furthermore, given that the O2 and P2 peptides were in the same aminoacidic position of the Ole e 7 and Pru p 3 aligned proteins, we performed their alignment comparison, with a result of three identities in a fragment of nine aligned amino acids. Moreover, as the O2 and P3 peptides were recognised by the sera pool of the three groups of patients, we also performed the sequence alignment comparison without finding any significant similarity. Finally, the search for sequences similar to the 11 residues of the P3 peptide using the BLAST tool provided 90% homology with Ara h 9, 80% with Mal d 3 and 73% with Cor a 8 and Jug r 3; in the case of the 11 residues of the O2 peptide of Ole e 7, a similarity between 80 and 100% was found with sequence peptides from some bacterial species such as Oscillospiraceae bacterium, Burkholderiales bacterium, Clostridia bacterium and Bacteroidetes bacterium HGW-Bacteroidetes-2.

## 4. Discussion

This study represents the first mapping of the Ole e 7 IgE-binding epitopes recognised by sera from Ole e 7-sensitised patients by a novel technique. This innovative approach also recognised the Pru p 3 epitopes previously described, confirming the method’s accuracy [39]. Additionally, this study provides the first description of the recognition of the Pru p 3 peptide “ISASTNCATVK” by sera from patients monosensitised to Ole e 7. Likewise, it reveals the recognition of the Ole e 7 peptide “KSALALVGNKV” by sera from patients monosensitised to Pru p 3. These results strongly suggest the possibility of cross-reactivity between the two proteins, particularly in those cases where BAT exhibited a higher positivity result with the non-sensitising allergen (e.g., those MON_OLE sensitised patients with higher amount of basophils reacting to Pru p 3). This finding underscores the potential for significant cross-reactivity, highlighting its importance in our analysis.

As previously mentioned, nsLTPs play a significant role in food allergies in Mediterranean countries, with Pru p 3 being the primary sensitising allergen in adult and paediatric populations [4,58]. In the southernmost parts of the Mediterranean Basin, sensitisation to Pru p 3 often coexists with sensitisation to Ole e 7. This observation raises a question: How can we provide evidence for cross-reactivity between two phylogenetically distant molecules?

In the present study, the BI sera pool recognised all three Pru p 3 peptides. However, the MON_PRU pool and, surprisingly, also the MON_OLE pool recognised the Pru p 3 P3 peptide “ISASTNCATVK” (Table 2). Epitope mapping of Pru p 3 has previously been performed by Garcia et al. by computer generation of 17 putative immunogenic peptides that they confronted with serum from seven patients monosensitised to Pru p 3 [39]. With a different approach, all patients recognised at least one of the same three epitopes corresponding to our P1, P2 and P3 peptides. In contrast, a similar study for Ole e 7 has not been conducted previously, leaving the characterisation of specific epitopes that could explain the hypothetical cross-reactivity between both allergens unresolved. Regarding the rOle e 7 mapping results of the present study, both MON_OLE and BI patients recognised the same peptide O2, “KSALALVGNKV”. Unexpectedly, MON_PRU patients could also recognise this rOle e 7 peptide (Table 2).

The search for sequence similarity between the two proteins, as anticipated from previous publications, revealed only a 32% identity, which is somehow insufficient according to the World Human Organization guidelines, which establish at least a 35% identity in a fragment of 80 linear amino acids with a 6–8 residue peptide to trigger a cross-reactivity. However, in structural epitopes, even though the involved amino acids do not require to be located consecutively, fragments of six to eight amino acids are sufficient [59], as in the case of IgE epitopes of Pru p 3 and other phylogenetically related fruit nsLTPs [6,18]. Particularly, the P3 “ISASTNCATVK” peptide exhibits 90% sequence identity with Ara h 9, 80% with Mal d 3, 73% with Jug r 3 and Cor a 8 and 71% with Lac s 1. Concerning the Ole e 7 O2 peptide “KSALALVGNKV”, it does not show significant sequence homology with any of the food (peanut, apple, walnut, hazelnut, lettuce and peach) or pollen (mugwort, wheat, plane tree and parietaria) nsLTPs, but it exhibits similarity ranging from 80% to 100% with proteins of bacterial origin that are present in the human microbiota, warranting further investigation. In summary, there is no apparent justification in the linear structure of these peptides, nor in the sIgE sensitisation profile of the three groups of patients for MON_PRU sera to recognise an Ole e 7 peptide or for MON_OLE sera to recognise a Pru p 3 peptide. Therefore, the cause for this phenomenon must be sought beyond the linear sequence [60].

Even though Pru p 3 and Ole e 7 exhibit limited sequence identity, certain residues associated with ligand binding are conserved or substituted by equivalent amino acids. For Pru p 3, these key residues correspond to Leu10, Ile14, Val17, Arg44, Leu51, Leu54, Ser55, Ala66 and Tyr79 [61], whereas, in Ole e 7, they are Leu18, Val33, Val36, Leu57, Leu72, Val80, Asp81, Leu82 and Leu85 [62]. On this point, antibodies against the sequence “CAGVK” of Ole e 7, surrounding the 36-valine site in the second alpha-helix (34-valine in Pru p 3), could potentially recognise the sequence “CATVK” of Pru p 3 (Figure 3A). The reason why MON_OLE patients did not recognise this area in Ole e 7 remains unclear. Structural rather than sequence similarity between the two proteins has been found even more, considering that the physical properties of amino acids determine the changes in the protein secondary structure [61,63]. The TM score obtained in the overlap model of the two proteins (Figure 3B), which considers global similarities rather than local discrepancies, was 0.8574, a remarkably high value if 1 is a perfect match.

In an experimental context, trypsin digestion disrupts peptide bonds of amino acids adjacent to arginine and lysine. When attempting to align a linear peptide resulting from trypsin digestion, it might be futile to seek perfect alignment, as digestion is an artificial process. Consequently, the epitope may exist in only part of the peptide, or the peptide could be affected by the cleavage site. In these circumstances, preformed antibodies against the sequence “CNCLKQLSASVPG” of Pru p 3, particularly in the region of Leu51, Leu54 and Ser55 within the third alpha-helix, might have the potential to recognise the sequence “CNCLKSALALVGN” of Ole e 7 (Figure 3A). This recognition could explain the binding to the peptide “KSALALVGNK” observed in the mapping of Ole e 7 [62].

The etiopathogenesis of allergies has been recently hypothesised as a multifactorial process that should not solely focus on the protein. In the case of allergy to nsLTPs, this theory assigns a crucial etiopathogenic role to the protein-borne ligand [64]. The hydrophobic cavity of nsLTPs could bind various ligands, including fatty acids or phospholipids. Binding to these ligands may influence the orientation of certain amino acid residues, inducing conformational changes that can impact the IgE-binding capacity [6]. This concept could elucidate why specific proteins and their associated lipids are allergenic in an “in vivo” context. Nevertheless, it is worth noting that the experiments in our study used recombinant proteins devoid of lipids; moreover, these proteins were digested into peptides, which discard the influence of this phenomenon in the binding capacity of patients’ sera.

The issue of cross-reactivity between Pru p 3 and Ole e 7 remains controversial, with evidence in both cases. One criterion used to discriminate the primary sensitising allergen in bisensitised patients is the result of inhibition tests [14,20,65]. In the ELISA and Western blot inhibition assays performed by Tordesillas et al. and Palacín et al. [20,65], a naturally isolated Ole e 7 protein was used, though only a 21-amino acid fragment was characterised. In the work of Oeo et al., as well as in our own work, a fully characterised recombinant Ole e 7 was employed [14]. On the other hand, another criterion used to discriminate the primary sensitising allergen in bisensitised patients is the level of sIgE to each allergen [17]. All bisensitised patients in our study showed higher sIgE values to Ole e 7 than to Pru p 3 (Table 1). In a recent paper, Asero et al. suggested that Pru p 3 may cross-react with Art v 3 and Pla a 3 as the sIgE levels to these nsLTPs were higher than those to their respective major pollen allergens, Art v 1 and Pla a 1/Pla a 2. This indicates that Pru p 3 acts as the primary sensitiser [17]. Similarly, in our study, the BI patients had higher levels of sIgE to Ole e 7 compared to Ole e 1, which supports the idea of Ole e 7 and Pru p 3 cross-reactivity in an area with extremely high olive pollen pressure (Table 1) [8,11,12]. In the study by Asero et al., the sIgE levels for several nsLTPs in Pru p 3 reactors increased over the years, except for Ole e 7 and Tri a 14, which remained unchanged. Additionally, while there was a significant correlation with a high coefficient between the levels of sIgE to Pru p 3 and to other nsLTPs, Ole e 7 showed a poor Spearman coefficient (0.399) [17]. Therefore, it seems that Ole e 7 deviates from the behaviour of other nsLTPs, highlighting its possible role as a primary sensitiser. In a recent paper, Asero et al. suggested that Pru p 3 may cross-react with Art v 3 and Pla a 3, as the sIgE levels to these nsLTPs were higher than those to their respective major pollen allergens, Art v 1 and Pla a 1/Pla a 2. This indicates that Pru p 3 acts as the primary sensitiser [17]. Similarly, in our study, BI patients had higher levels of sIgE to Ole e 7 compared to Ole e 1, which supports the idea of Ole e 7 and Pru p 3 cross-reactivity in an area with extremely high olive pollen pressure (Table 1) [8,11,12]. In the study by Asero et al., the sIgE levels for several nsLTPs in Pru p 3 reactors increased over the years, except for Ole e 7 and Tri a 14, which remained unchanged. Additionally, while there was a significant correlation with a high coefficient between the levels of sIgE to Pru p 3 and to other nsLTPs, Ole e 7 showed a poor Spearman coefficient (0.399) [17]. Therefore, it seems that Ole e 7 deviates from the behaviour of other nsLTPs, highlighting its possible role as the primary sensitiser. Regarding the sIgE sensitisation panel studied in the present research, a striking finding was the reactivity profile of the sera in each group. Among the nsLTPs studied, MON_OLE sera only reacted to Ole e 7, whereas BI and MON_PRU sera reacted to almost all nsLTPs (Table 1). Therefore, it could be hypothesised that a sensitisation to Ole e 7 occurs differently in MON_OLE patients compared to BI patients.

The main limitation of this study is the restricted number of patient sera from each profile used in the research, which may have led to an underestimation of the number and the role of the Ole e 7 epitopes. Nevertheless, the levels of sIgE to Ole e 7 in MON_OLE patients were high enough for detecting existing epitopes, whereas those specific for Pru p 3 in the MON_PRU patients were not as high, potentially leading to an under-representation of epitope identification. Additionally, a sera pool was used instead of individual samples from each group, which may have masked the heterogeneity in the antibody response between different patients. However, this approach was used to integrate all potential heterogeneity and is consistent with other studies that used serum pools to search for Pru p 3 epitopes [39,40], although using different techniques. Another limitation is related to the epitope mapping method, as trypsin digestion could potentially destroy sIgE-binding residues. To address these limitations, future studies are essential to validate these initial findings; these could include a large cohort of patients, the use of individual patient sera and alternative epitope mapping methods that do not rely on trypsin digestion, such as employing synthetic peptide, as well as inhibition studies using the described peptides, as other groups accomplished with Pru p 3 or other allergen epitope identification [36,37,38,39,40]. Since this is the first mapping of the Ole e 7 epitopes, there are no existing data to contrast the results, although it would be interesting to prove if these findings are replicable in individuals from the Mediterranean Basin, where the epidemiological and geographic conditions are similar.

## 5. Conclusions

This study unveils, for the first time, two sIgE epitopes within the nsLTP Ole e 7 allergen using a novel approach and confirms those described in the Pru p 3 allergen. Interestingly, the sera pool from individuals solely sensitised to Ole e 7 based on their sIgE profile was able to recognise a peptide derived from rPru p 3, which was supported by the ability of their basophils to degranulate in the presence of Pru p 3. Conversely, the sera pool from individuals monosensitised to Pru p 3 recognised a peptide from Ole e 7. This phenomenon occurs despite the lack of significant linear sequence similarity between the two proteins. This fact suggests that the structural homology supported by a predicted three-dimensional alignment with a very high TM score and the physical properties of shared key ligand-binding residues may play a crucial role in the potential cross-reactivity between Ole e 7 and Pru p 3 nsLTPs. Moreover, this finding reinforces the complex nature of allergen interactions and underscores the importance of considering structural features beyond sequence similarity when understanding cross-reactivity in nsLTPs.

## Figures and Tables

**Table 1:** Specific IgE profiles to different allergens from the sera used in the epitope mapping and Basophil Activation Test (BAT) results. The selection of quantified allergens was based on relevant food and inhaled nsLTP allergens in our geographical area, along with a non-nsLTP one from olive pollen, Ole e 1, of great importance for olive-sensitised patients (MON_OLE and BI). The BAT results showed the percentage of degranulated basophils in response to rOle e 7 and rPru p 3 stimuli; a test was considered positive when the percentage of degranulated basophils was greater than 10%.

	sIgE ^1^										BAT ^2^	
	Ole e 1 ^3^	Ole e 7 ^4^	Pru p 3 ^5^	Mal d 3 ^6^	Art v 3 ^7^	Tri a 14 ^8^	Jug r 3 ^9^	Cor a 8 ^10^	Ara h 9 ^11^	Par j 2 ^12^	rOle e 7 (10 µg/mL)	rPru p 3 (10 µg/mL)
1. MON_OLE ^13^	0.02	**149**	0.03	0.04	0.06	0.02	0.05	0.02	0.02	0.04	**60.6**	**18.6**
2. MON_OLE	**1.26**	**69.7**	0.01	0.02	0.02	0	0.01	0	0.01	0.02	**84.7**	**78.8**
3. MON_OLE	**14.3**	**192**	0.05	0.06	0.04	0.02	0.08	0.02	0.03	0.04	**10.7**	**14.1**
4. MON_PRU ^14^	NA ^15^	0.1	**6.32**	**6.13**	**1.48**	**1.75**	**1.81**	**5.35**	**6.07**	0.03	5.3	**22.7**
5. MON_PRU	NA	0.33	**82.8**	**59.3**	**47**	**6.68**	**50.9**	**29.1**	**30.1**	0.28	3.5	**69.0**
6. MON_PRU	NA	0.08	**10.8**	**11.2**	**0.63**	0.28	**5.22**	**3.79**	**5.75**	0.02	4.8	**46.6**
7. BI ^16^	0	**39.2**	**29.5**	**26.1**	**17.5**	**17.7**	**19.3**	**6.43**	**26.4**	0.01	**36.5**	**48.1**
8. BI	98.1	**392**	**202**	**156**	**93.2**	**42.2**	**69.1**	**52**	**135**	0.12	**17.5**	**39.6**
9. BI	**10.1**	**30.7**	**23.3**	**20.9**	**31.4**	**6.42**	**18.8**	**7.77**	**20.4**	0.1	**69.7**	**49.8**

sIgE ^1^, specific IgE. BAT ^2^, Basophil Activation Test: percentage (%) of degranulated basophils. Ole e 1 ^3^, major allergen of olive pollen. Ole e 7 ^4^, olive pollen nsLTP. Pru p 3 ^5^, peach nsLTP. Mal d 3 ^6^, apple nsLTP. Art v 3 ^7^, mugwort nsLTP. Tri a 14 ^8^, wheat nsLTP. Jug r 3 ^9^, walnut nsLTP. Cor a 8 ^10^, hazelnut nsLTP. Ara h 9 ^11^, peanut nsLTP. Par j 2 ^12^, parietaria nsLTP. MON_OLE ^13^, serum from patients monosensitised to Ole e 7. MON_PRU ^14^, serum from patients monosensitised to Pru p 3, negative results for Ole e 7. NA ^15^, not assessed. BI ^16^, serum from patients sensitised to Ole e 7 and Pru p 3. Bold numbers indicate values that exceeded the positivity threshold.

**Table 2:** IgE-binding epitopes of rPru p 3 and rOle e 7.

	Bound Ole e 7 Epitopes ^1^	Bound Pru p 3 Epitopes ^2^
MON_OLE ^3^ serum pool	O1: KLTSCVSYLDDKS O2: KSALALVGNKV	P3: ISASTNCATVK
MON_PRU ^4^ serum pool	O2: KSALALVGNKV	P3: ISASTNCATVK
BI ^5^ serum pool	O2: KSALALVGNKV	P1: NVNNLAR P2: QLSASVPGVNPNNAAALPGK P3: ISASTNCATVK

^1,2^ Sequenced peptides obtained from immunocapture and LC-MS analysis for each group of patients. MON_OLE ^3^, patients monosensitised to Ole e 7. MON_PRU ^4^, patients monosensitised to Pru p 3. BI ^5^, patients sensitised to Ole e 7 and Pru p 3.

## Data Availability

The data presented in this study are available on request from the corresponding author due to ethical reasons.

## Supplementary Materials

The following supporting information can be downloaded at https://www.mdpi.com/article/10.3390/nu16172869/s1: Figure S1: Basophil degranulation plots of individual blood samples stimulated with PBS, fMLP, 10 μg/mL of rOle e 7 and rPru p 3. BAT A-C corresponded to MONOLE patients, D-F to MONPRU (in the case of patient D, a smaller number of events was acquired), and G-I to BI patients. Plots 1 and 2 showed negative and positive controls, respectively. rOle e 7-stimulated BAT is represented with number 3 and rPru p 3-stimulated BAT with number 4. Percentage of basophil degranulation with both allergens are shown in Table 1; Table S1: Epitope mapping output data showing relative quantification of each peptide.
