# Supplementary material for: Pollen-Food Allergy Syndrome: From Food Avoidance to Deciphering the Potential Cross-Reactivity between Pru p 3 and Ole e 7
Source: Nutrients. 2024 Aug 27;16(17):2869. doi: 10.3390/nu16172869 (PMC11396898; doi:10.3390/nu16172869)
Supplement: Supplementary file 1 [file nutrients-16-02869-s001.zip › nutrients-3124575-supplementary materials.pdf]

Supplementary Material

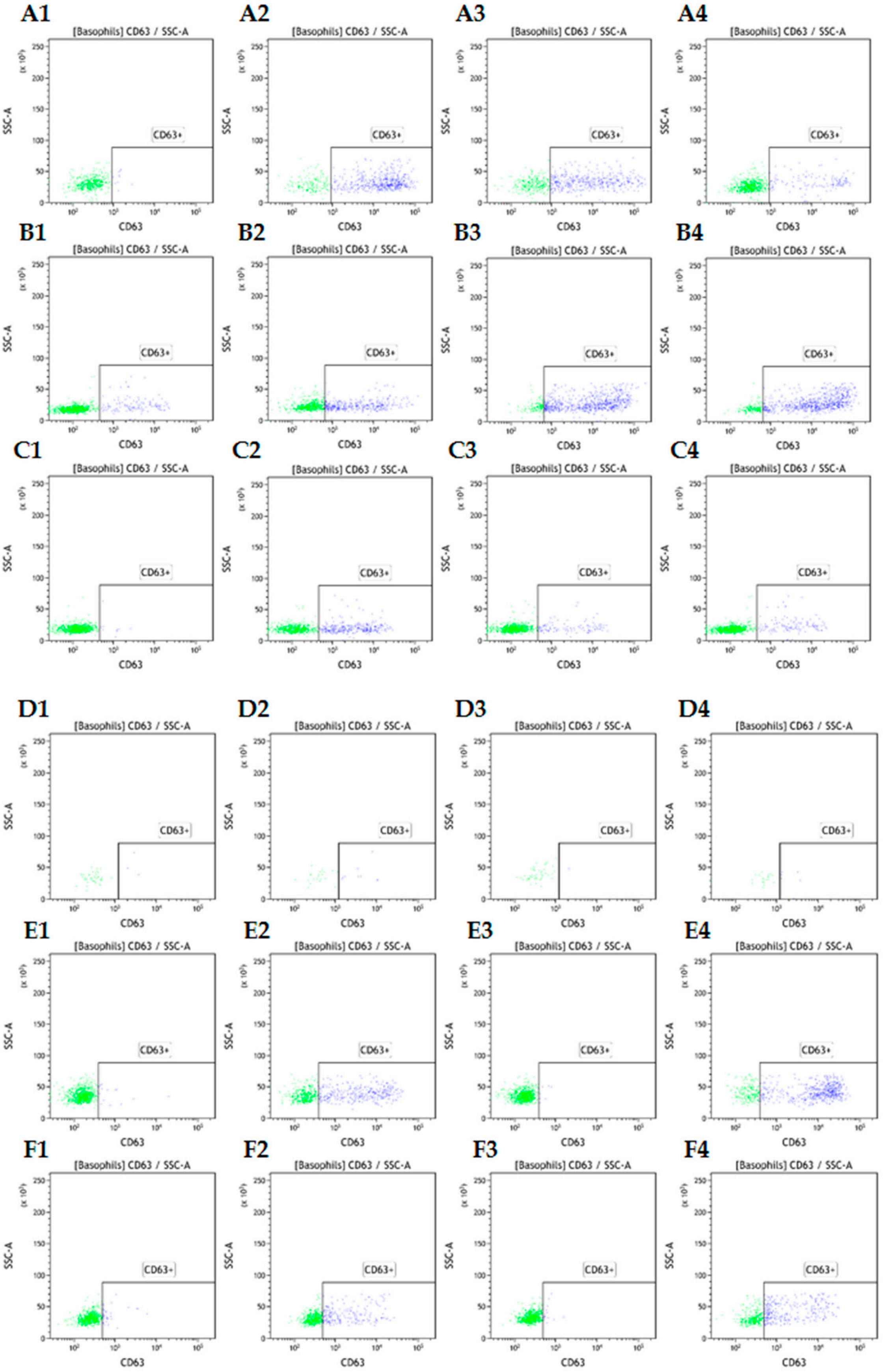

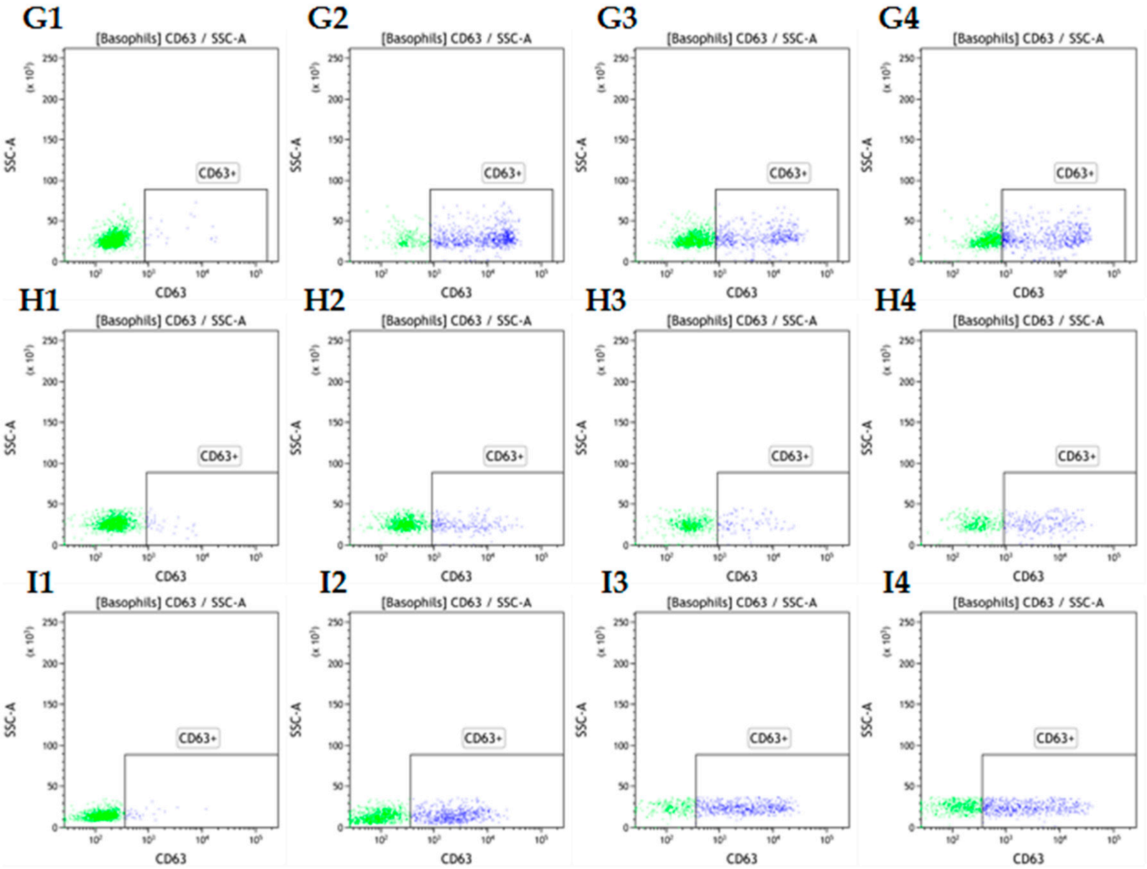

Figure S1. Basophil degranulation plots of individual blood samples stimulated with PBS, fMLP, 10 µg/mL of rOle e 7 and rPru p 3. BAT **A-C** corresponded to MONOLE patients, **D-F** to MONPRU (in the case of patient D, a smaller number of events was acquired), and **G-I** to BI patients. Plots 1 and 2 showed negative and positive controls, respectively. rOle e 7-stimulated BAT is represented with number 3 and rPru p 3-stimulated BAT with number 4. Percentage of basophil degranulation with both allergens are shown in Table 1.

Table S1. Epitope mapping output data showing relative quantification of each peptide.

Protein	Samples	Peptide	Intensity[1]	Ions[2]
Ole e 7	Non-allergic control	ND[3]	ND	ND
	BI[4] serum pool	KSALALVGNKV	3.49E-02	9/16
	MON_OLE[5] serum pool	KLTSCVSYLDDKS	4.27E-03	11/20
		KSALALVGNKV	0.0004385	9/16
	MON_PRU[6] serum pool	KSALALVGNKV	4.72E-02	9/16
Pru p 3	Non-allergic control	ND	ND	ND
	BI serum pool	QLSASVPGVNPNNAAALPGK	3.68E-10	19/38
		NVNNLAR	0.01936	4/12
		ISASTNCATVK	4.61E-07	13/20
	MON_OLE serum pool	ISASTNCATVK	4.84E-06	11/20
	MON_PRU serum pool	ISASTNCATVK	1.58E-05	10/20

[1]Intensity expressed as arbitrary units from raw data; [2]Ions: number of ions matched for each detected peptides; [3]ND: not detectable; BI[4], patients sensitised to Ole e 7 and Pru p 3; MON_OLE[5], patients monosensitised to Ole e 7. MON_PRU[6], patients monosensitised to Pru p 3.
