# Supplementary figures and images for: Pollen-Food Allergy Syndrome: From Food Avoidance to Deciphering the Potential Cross-Reactivity between Pru p 3 and Ole e 7
Source: Nutrients. 2024 Aug 27;16(17):2869. doi: 10.3390/nu16172869 (PMC11396898; doi:10.3390/nu16172869)

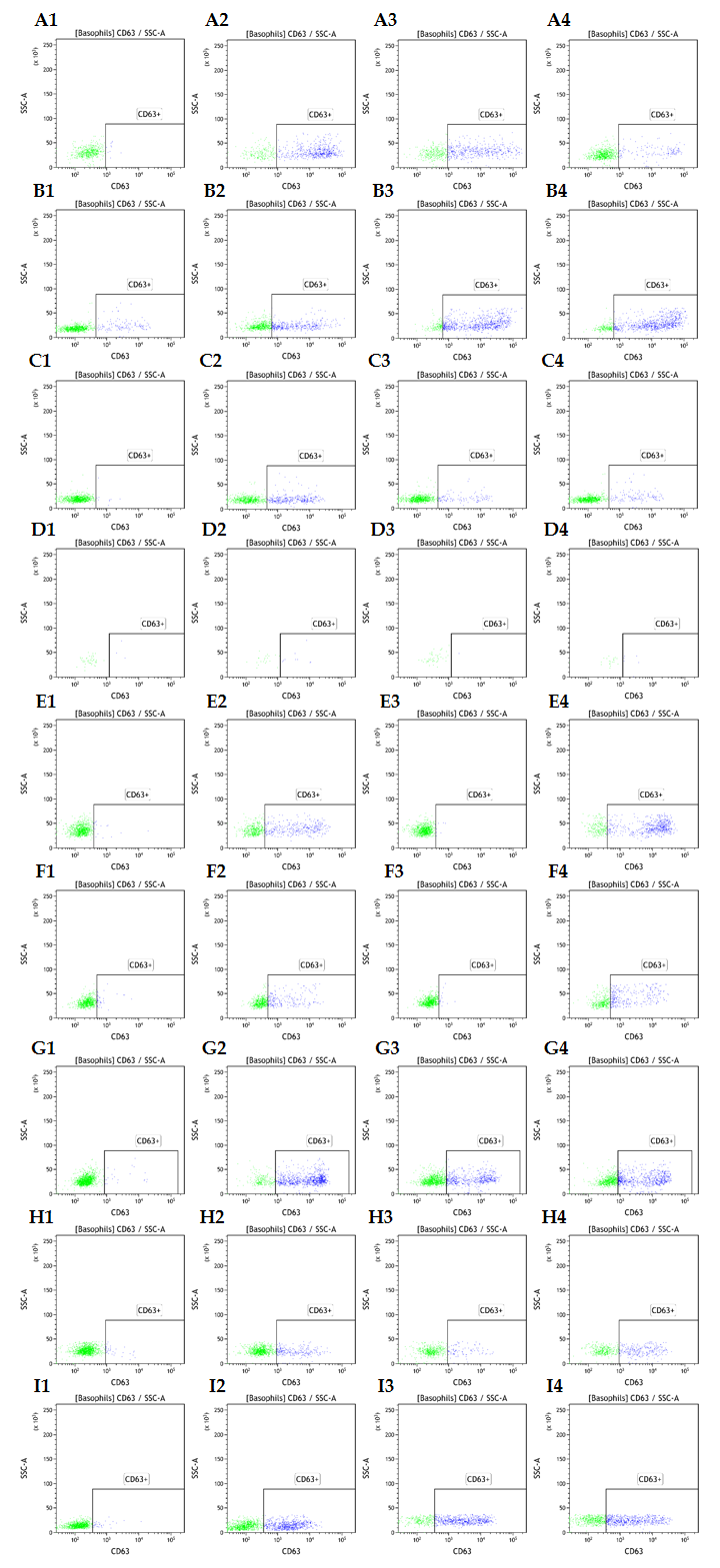

Supplement: Supplementary file 1 [file nutrients-16-02869-s001.zip › Figure S1.png]
